# Successful surgical management of massive hemoretroperitoneum caused by spontaneous rupture of retroperitoneal lymph node metastases in a patient with advanced mixed germ cell tumor: a COVID-19 pandemic-related surgical challenge

**DOI:** 10.1186/s40792-023-01593-z

**Published:** 2023-02-06

**Authors:** Konstantinos Vasiliadis, Chrysanthi Simou, Anastasios Tzotzou, Nikolaos Kalinderis, Dimitrios Valoukas, Elsa Pazarli, Paulos Drakontaidis, Christos Papavasiliou

**Affiliations:** 1 Surgical Department, Papageorgiou General Hospital of Thessaloniki, Thessaloniki, Greece; 2 2Nd Department of Urology, Aristotle University of Thessaloniki, Thessaloniki, Greece; 3 Department of Medical Oncology, Aristotle University of Thessaloniki, Thessaloniki, Greece; 4 Department of Pathology, Papageorgiou General Hospital of Thessaloniki, Thessaloniki, Greece; 5 Radiology Department, Papageorgiou General Hospital of Thessaloniki, Thessaloniki, Greece

**Keywords:** Testicular cancer, Retroperitoneal lymph node metastasis, Hemoperitoneum, The Cattell–Braasch maneuver, Retroperitoneal hematoma, Mixed germ cell tumor

## Abstract

**Background:**

Spontaneous rapture of a germ cell tumor (GCT) metastases causing massive hemoretroperitoneum in a patient without choriocarcinoma component who has not received previous systemic chemotherapy is an exceedingly rare event. In such a devastating case scenario, a high index of clinical suspicion for early diagnosis and appropriate management is crucial.

**Case presentation:**

We report on a 25-year-old male patient with a 4-month history of orchiectomy for testicular GCT (tGCT), who presented in the emergency department with acute abdomen and hemodynamic instability. Urgent computed tomography scan depicted a retroperitoneal mass measuring approximately 13 × 11.4 × 15 cm and massive intraperitoneal hemorrhage. Hemoperitoneum caused by spontaneous rapture of the metastatic retroperitoneal mass was suspected. COVID-19 pandemic-related deviation from the oncologic surveillance standards combined with COVID-19-related patient’s emotional distress and self-neglect had led to loss of opportunity for appropriate adjuvant chemotherapy, obviously leading to the development of this devastating complication. An emergency, surgical exploration was decided. The bleeding mass was adequately exposed following a Cattell–Braasch maneuver and active bleeding was controlled by a challenging resection of approximately 80% of the lymph node mass volume. Pathological evaluation of the specimen revealed teratoma with low volume of yolk sac tumor component and extensive necrosis, findings compatible with the patient’s history. Postoperative recovery was uneventful, followed by early start of adjuvant chemotherapy. Two years after the operation the patient is doing well with no evidence of recurrent disease.

**Conclusions:**

Massive hemoperitoneum is a devastating event that exceedingly rarely can complicate the clinical course of patients with advanced tGCT. Emergency surgical intervention is usually necessary however, sound judgement and careful surgical techniques are required for a positive and uneventful outcome. During COVID-19 pandemic, first-line medical personnel push their limits further not only to ensure health care services standards but also, to manage unpredictable, life-threatening cancer-related complications, associated with COVID-19-related deviation from appropriate oncologic surveillance and care.

## Background

Testicular germ cell tumors (tGCT) are common malignant lesions among young men aged 20–45 years. They often spread via lymphatics or hematogenously, and they typically metastasize to the retroperitoneal lymph nodes, lungs, liver, bones or brain. [1]. Germ cell tumor metastases are frequently present at diagnosis [2] increasing the risk of adverse events. However, major complications related to GCT metastatic sites are very rare [3]. In this regard, there are scanty data in the literature referring to metastatic germ cell tumors in the retroperitoneal lymph nodes as source of massive hemorrhage [3–6]. Actually, life-threatening bleeding can rarely complicate the clinical course of patients with metastatic tGCT. Most of the reported cases of hemoperitoneum and/or hemoretroperitoneum related to testicular cancer, refer to spontaneous rupture of hepatic or retroperitoneal lymph node metastases of tumors containing large volume of choriocarcinoma elements after the initiation of chemotherapy [7].

The present report describes the successful surgical management of an exceedingly rare case of hemoperitoneum caused by spontaneous rapture of tGCC retroperitoneal lymph node metastases in a patient without choriocarcinoma component who has not received previous systemic chemotherapy. Active bleeding was uneventfully controlled by a challenging surgical resection of approximately 80% of the metastatic lymph node mass volume.

Regardless of the rarity and favorable outcome of the present case, it should be emphasized that this exceptional complication is actually a consequence of COVID-19 pandemic-related disruption in oncologic surveillance, which inevitably resulted in loss of opportunity for appropriate adjuvant therapy and the development of this devastating complication.

## Case report

**Fig. 1:**
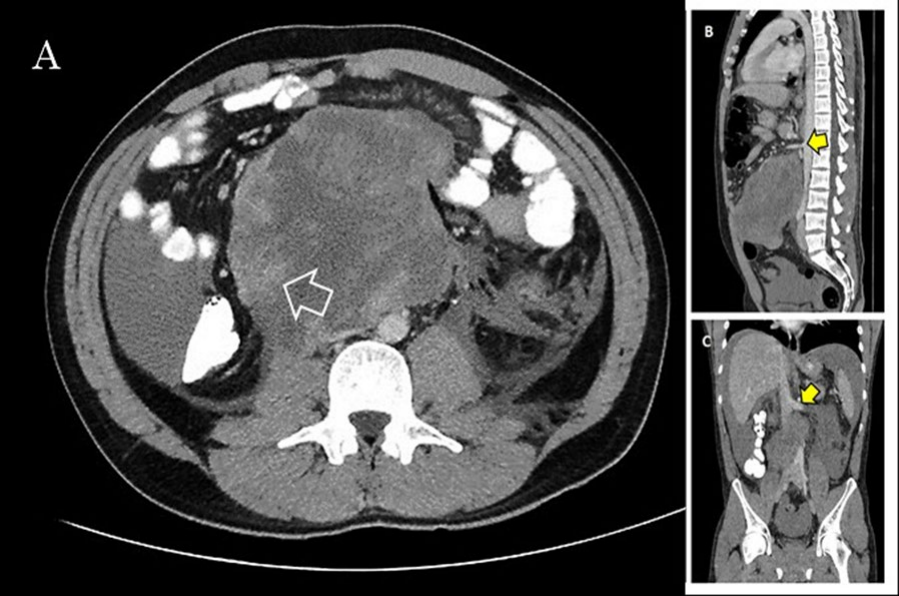
Preoperative abdominal contrast-enhanced CT showing **A** the large 13 × 11.4 × 15 cm retroperitoneal mass extending to the preaortic region below the renal veins. A liquefied and necrotic center with hyperdense areas inside the lymph node metastatic mass is clearly depicted (marked with a white arrow) on preoperative CT suggesting bleeding. **B** Sagittal view of the tumor and its relations with the superior mesenteric artery (yellow arrow) and **C** coronal view of the tumor and its relations with the left renal vein (yellow arrow), in addition to a significant amount of free intraperitoneal fluid suggestive of hematic content

A 25-year-old male patient presented to the Emergency Department complaining of sudden onset of diffuse abdominal pain which radiated to his back, associated with nausea and numerous episodes of vomiting and diarrhea. On physical examination he appeared diaphoretic and quite uncomfortable, while his vital signs were suggestive of early stages of hypovolemic shock. He had tachycardia (regular rhythm, 120 bpm) with however, normal blood pressure. Abdominal distention, diffuse abdominal tenderness and a palpable mass in the periumbilical region were also evident. Initial laboratory tests revealed high white blood cell count (28.4 × 10^9^/L), relatively low hemoglobin levels (10.4 g/dL), moderate hyperglycemia (225 mg/dL), slightly elevated urea and creatinine levels (60 mg/dL and 2.26 mg/dL, respectively) and normal lactate dehydrogenase levels (140 U/L). An urgent contrast-enhanced computed tomography (CT) scan of the abdomen and pelvis depicted a large 13 × 11.4 × 15 cm retroperitoneal mass extending to the preaortic region below the renal veins in addition to a significant amount of free intraperitoneal fluid suggestive of hematic content (Fig. 1). In addition, the mass caused partial obstruction of the right ureter and mild ipsilateral hydronephrosis.

The patient had a history of a radical right orchiectomy 4 months ago, for a malignant non-seminoma mixed GCT. The resected tumor had a diameter of 11 cm, consisting of teratoma (45%), embryonal cancer (40%), seminoma (10%) and yolk sac tumor (5%), and was classified as pT2 according to the 8th edition of AJCC cancer staging manual [8]. His pre-orchiectomy CT staging, showed 2 focal liver lesions, compatible with hemangiomas and an enlarged (11 mm in diameter) preaortic lymph node at the level of aortic bifurcation. Preoperative levels of alpha-fetoprotein (AFP) and beta-human chorionic gonadotropin (HCG) were normal (β-HCG < 1 mIU/L and α-FP = 6 ng/mL). A preoperative brain magnetic resonance imaging (MRI) showed no signs of metastatic disease. Pathologic evaluation of the resected specimen classified the tumor as stage II, under the 8th edition of AJCC cancer staging manual [8], and the prognostic criteria of the International Germ Cell Cancer Collaborative Group (IGCCCG) [9] predicted a favorable disease prognosis. However, COVID-19 pandemic-related deviation from oncologic surveillance and care standards combined with COVID-19-related patient’s emotional distress and self-neglect, had led to loss of opportunity for timely and appropriate adjuvant chemotherapy, obviously leading to the development of this devastating complication [10].

The patient was urgently admitted to the intensive care unit (ICU) for hemodynamic resuscitation. Vital signs at arrival in the ICU were pulse of 130 beats per minute, blood pressure 95/60 mm Hg, and hemoglobin level of 8.6 g/dL, suggesting a deteriorating hypovolemic shock. The patient was resuscitated with 2 L of intravenous fluids, 2 units of packed red blood cells, 2 units of fresh frozen plasma and vasopressors. He did not respond satisfactory to fluid load, while vasopressor needs were increased, with post-transfusion hemoglobin of 8.1 g/dL, and ABGs lactate levels 3.1 mmol/L. However, apart from detailed description of resuscitation requirements, the most decisive clinical sign was the progressively worsening abdominal distension of the patient, indicating an ongoing, uncontrolled intraperitoneal hemorrhage. This, combined with ICU monitoring data, laboratory values and computed tomography findings (massive active hemoretroperitoneum with a significant amount hemoperitoneum) led to the decision of an emergency exploratory laparotomy.

The peritoneal cavity was immediately accessed through a midline laparotomy. Massive hemoperitoneum was evident, necessitating evacuation of 2.5 L of blood, in order to identify the source of bleeding. A massive retroperitoneal hematoma surrounding a bleeding retroperitoneal lymph node metastatic necrotic mass was evident. Shearing and infiltrating forces exerted from the bleeding retroperitoneal metastatic mass, teared in several sites the posterior peritoneum, through which the blood entered the peritoneal cavity causing massive hemoperitoneum.

**Fig. 2:**
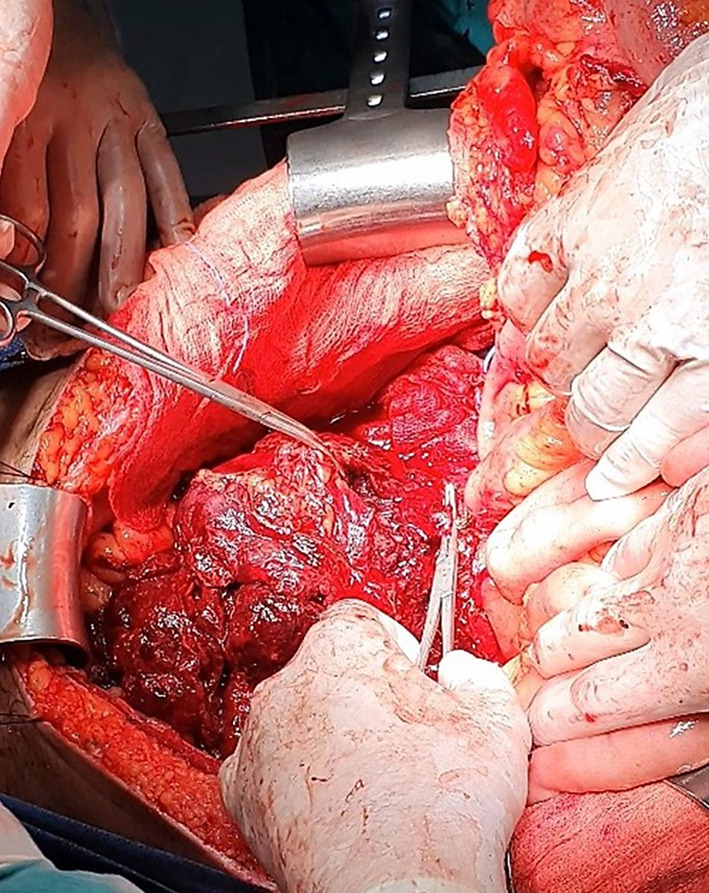
Intraoperative photograph. Exposure of the bleeding mass following completion of the Cattell–Braasch maneuver. Αdhesions between the lymph node metastatic mass and right spermatic vessels necessitated their en bloc resection (right spermatic vessels clamped with Kelly forceps)

Following a right-sided medial visceral rotation maneuver (the Cattell–Braasch maneuver) consisting of extensive lateromedial-craniomedial detachment and complete mobilization of the right hemi-colon and small bowel mesentery from the retroperitoneum along the fusion fascia of Toldt [11] the retroperitoneal mass was immediately accessed (Fig. 2). Given that the Cattell–Braasch maneuver enabled adequate exposure of the mass, performing additionally a Mattox maneuver was deemed unnecessary.

**Fig. 3:**
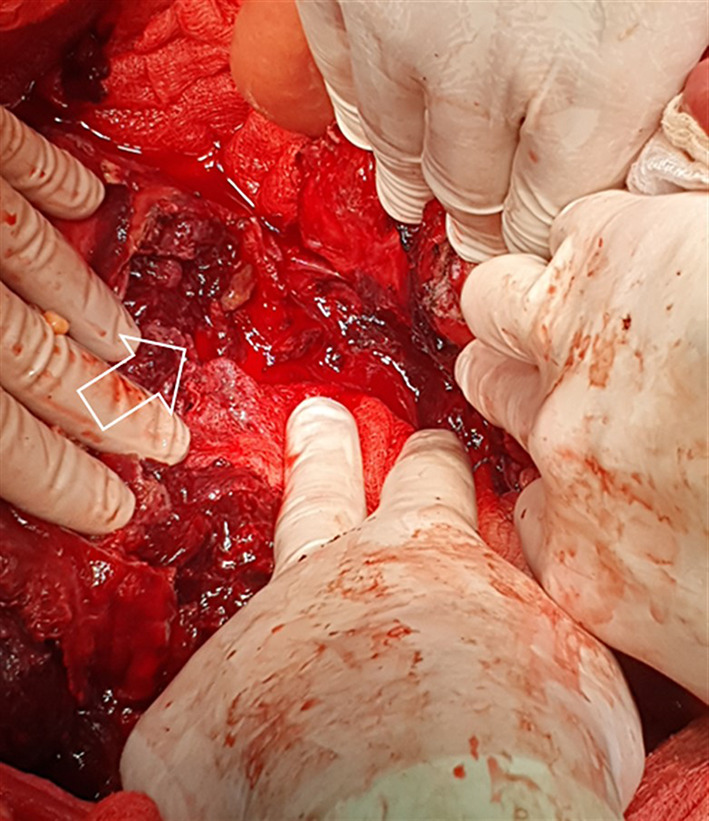
Intraoperative photograph. The right part of the infracolic centromedial retroperitoneum is completely invaded by the mass which is actively bleeding from its necrotic surface areas (arrow)

A huge lymph node metastatic mass was evident located in the centromedial retroperitoneum extending from the renal veins to the aortic bifurcation. The mass was very soft and fragile with necrotic surface areas, which were actively bleeding (Fig. 3). Additionally, the mass was displacing small and large bowel towards the left side and was densely adherent to the right ureter and the horizontal part of the duodenum. The main part of the metastatic tumor volume was located in the right part of the infracolic centromedial retroperitoneum on the right side of the aorta along the aortocaval lymph nodes. Blood extravasation infiltrating surrounding tissues, profound bleeding and dislocated viscera, made safe surgical dissection along avascular embryological planes fraught with pitfalls. Notwithstanding, careful dissection enabled complete exposure of the bleeding mass. Following safely identification and safeguarding of great vessels, critical tributaries and branches, control of the hemorrhage was finally achieved by a challenging resection of approximately 80% of the metastatic lymph node mass volume. Part of the mass invading the right ureter and the horizontal part of the duodenum remained in situ. Total intraoperative blood loss was evaluated to be around 4 L, while the patient received 4 units of fresh frozen plasma and 4 units of red blood cells.

**Fig. 4:**
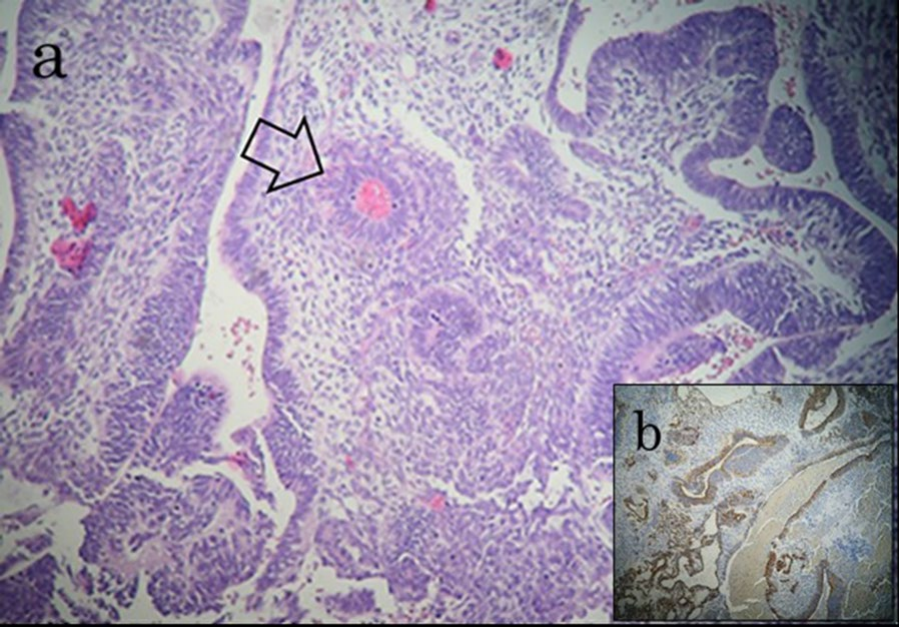
**a** Histological section showing immature teratoma composed of undifferentiated cells with foci of primitive neuroectodermic differentiation forming immature neuroepithelial tissue (arrow), in a cellular stroma (hematoxylin and eosin, 20 × magnification). **b** Immunochemical results demonstrated positivity for S 100 protein and glial fibrillary acidic protein markers, compatible with primitive neuroectodermal tissue (metastases of the teratoma component of the primary mixed GCT), (20 × magnification)

**Fig. 5:**
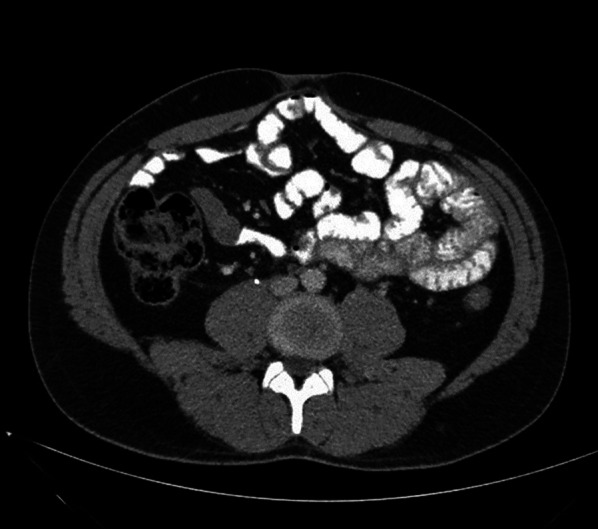
Abdominal contrast-enhanced CT 2 years after the operation showing no residual disease

Pathological evaluation of the surgical specimen revealed morphological features of immature teratoma composed of undifferentiated cells with foci of primitive neuroectodermic differentiation forming immature neuroepithelial tissue and extensive necrosis, findings compatible with the patient’s history of mixed tGCT (metastases derived from the teratoma component of the primary mixed GCT) (Fig. 4). Postoperative recovery was uneventful, and was followed by early start of adjuvant chemotherapy without adverse events with the patient still hospitalized, with three cycles of cisplatin/etoposide followed by a cycle of bleomycin/cisplatin/etoposide. The patient consented to a regular follow up according to recent standards of oncologic surveillance and care. Two years after the operation the patient is doing well with no evidence of recurrent disease (Fig. 5).

## Discussion

In response to the increase prevalence of the coronavirus disease at the end of October 2020 in Greece, several restrictive measures were accommodated, which unavoidably have had drastic impacts on healthcare system services. In particular, COVID-19 pandemic-related deviation from the standard oncologic continuum has profoundly impacted cancer care leading to delay or even loss of opportunity for appropriate treatment [12]. In the present case, COVID-19 pandemic-related deviation from oncologic surveillance and care standards combined with COVID-19-related patient’s emotional distress and self-neglect had led to loss of opportunity for timely and appropriate adjuvant chemotherapy. Therefore, considering the chemosensitivity of GCTs, it can be argued, that this devastating complication could have been avoided in the event that the standards of oncologic care were appropriately met [13]. During the COVID-19 pandemic era, oncologic surveillance and care disruption resulted in higher rates of serious, life-threatening cancer-related complications [10] requiring, as in the present case challenging and demanding surgical management.

Notwithstanding, severe and devastating complications related to testicular cancer metastases, such as massive retroperitoneal and/or intraperitoneal hemorrhage, are exceedingly rare. Most of the reported cases are related to rupture of liver metastases or retroperitoneal metastatic masses after the onset of chemotherapy. These cases are mainly associated with choriocarcinoma syndrome, [14–16] which refers to diffuse germ cell tumor burden with high volume choriocarcinomatous elements, that manifests as life-threatening hemorrhage at metastatic sites in whichever organ system is involved and is associated with a poor outcome [7, 17]. In these devastating cases, chemotherapy treatment may represent the most significant triggering mechanism for tumor lysis and massive bleeding [7]. Of note is that, in the present case massive hemoretroperitoneum developed before the initiation of systemic chemotherapy and the primary tumor had no choriocarcinomatous elements.

Lymphatic spread of testicular germ cell cancer follows usually a predictable pattern, metastasizing most commonly in the retroperitoneal lymph nodes. Specifically, right testicle GCT often metastasize to the interaortocaval nodes while left-sided tumors metastasize to the preaortic and paraortic lymph nodes [18, 19]. This is in line with the site of the main burden of metastatic tumor volume in the present case, which was located in the right part of the infracolic centromedial retroperitoneum on the right side of the aorta along the aortocaval lymph nodes.

Massive spontaneous hemoperitoneum secondary to metastatic masses usually necessitates an emergency surgical exploration however, it should be taken into account that it demands surgical skills and expertise and that it can be associated with dismal intra- and post-operative complications and considerable morbidity. Therefore, accurate diagnosis, sound judgement and careful surgical techniques are required for a positive and uneventful outcome. Indeed, caution is needed considering that retroperitoneal bleeding may also develop as a result of laceration of major retroperitoneal vessels during systemic therapy [5].

Special attention is warranted to the usefulness of the Cattell–Braasch maneuver in these extreme emergencies. This master surgical technique succeeds safe, immediate and adequate exposure of the retroperitoneum in salvage and damage control operations [20]. Particularly, the right-sided medial visceral rotation maneuver can be used to expose the subhepatic inferior vena cava, the right kidney and its pedicle, the inframesocolic aorta up to its bifurcation, the superior mesenteric artery, the superior mesenteric vein, the inferior mesenteric artery, the right iliac vessels, the right ureter, the duodenum and the head of the pancreas [20]. In the present case, the retroperitoneal mass was safely and immediately accessed following the Cattell–Braasch maneuver exposing adequately the bleeding metastatic mass.

Urgent surgical exploration has been usually performed in a handful of similar cases reported in the literature. Komori et al. [4] reported on the surgical management of hemoperitoneum caused by spontaneous rapture of a mixed germ cell tumor metastatic retroperitoneal lymph node mass penetrating the retroperitoneum between the ligament of Treitz and the inferior mesenteric vein. The patient had considerably high β-HCG and α-FP levels. Despite significant intraoperative blood loss approximately 2 L, complete hemostasis has been accomplished with the patient withstanding the surgical procedure. Unfortunately, the patient died one month later because of massive gastrointestinal hemorrhage [4].

Moore et al. [3] reported on a patient with a mixed nonseminomatous GCT who developed massive hemoperitoneum secondary to a ruptured retroperitoneal metastatic lymph node mass, composed of more than 95% of choriocarcinoma. With the expense of 10 L of intraoperative blood loss, surgical exploration and resection of 90% of the mass accomplished complete hemostasis. Filho et al. [6] reported on a man with non-seminoma germ cell tumor who presented with massive spontaneous retroperitoneal hemorrhage. As in the present case, the patient did not receive previous systemic treatment and the primary tumor had no choriocarcinoma component. Contrary to the previously mentioned case reports and to the present case, the patient reported by Filho et al. [6] was managed without surgical treatment, with good clinical outcome. Certainly, conservative treatment should always be considered initially in the management of similar cases, on condition that the patient responds well to the resuscitation measures and remains hemodynamically stable, without the need of vasoactive agents and/or blood substitutes**.** If this is not the case, surgical approach should be considered. In the present case, despite hemodynamic resuscitation, the patient remained hemodynamically unstable, which led to the decision of an emergency, surgical exploration. Nevertheless, it should always be kept in mind that this devastating clinical scenario represents a lethal cancer-related complication and appropriate management should be individualized, given that there are no evidence-based guidelines.

In conclusion, spontaneous rapture of a germ cell cancer retroperitoneal lymph node metastasis in a patient with tGCT without choriocarcinoma component who has not received previous systemic chemotherapy is exceedingly rare. Emergency surgical intervention is usually necessary however, dismal intra- and post-operative complications can frequently occur. Sound judgement, experience and careful surgical techniques are required for a positive and uneventful outcome. During COVID-19 pandemic, first-line medical personnel push their limits not only to ensure healthcare services standards, but also to manage unpredictable, life-threatening cancer-related complications associated with COVID-19-related deviation from appropriate oncologic surveillance and care.

## Data Availability

Not applicable.

## Abbreviations

GCT: Germ cell tumor
tGCT: Testicular germ cell tumor
CT: Computed tomography
AJCC: American Joint Committee on Cancer
β-HCG: Beta-Human chorionic gonadotropin
α-FP: Alpha-fetoprotein
MRI: Magnetic resonance imaging
IGCCCG: International Germ Cell Cancer Collaborative Group
ICU: Intensive care unit
ABGs: Arterial blood gases
